# Application of Covalent Organic Porous Polymers-Functionalized Basalt Fibers for in-Tube Solid-Phase Microextraction

**DOI:** 10.3390/molecules25245788

**Published:** 2020-12-08

**Authors:** Qiong Jiang, Peng Xu, Juanjuan Feng, Min Sun

**Affiliations:** 1 College of Plant Protection, Gansu Agricultural University/Biocontrol Engineering Laboratory of Crop Diseases and Pests of Gansu Province, Lanzhou 730070, China; xupeng@gsau.edu.cn; 2 Key Laboratory of Interfacial Reaction & Sensing Analysis in Universities of Shandong, School of Chemistry and Chemical Engineering, University of Jinan, Jinan 250022, China; chm_fengjuanjuan@ujn.edu.cn

**Keywords:** covalent organic porous polymers, basalt fibers, in-tube solid-phase microextraction, online analysis, estrogens

## Abstract

To establish an online analytical method towards estrogenic pollutants, a covalent organic porous polymer (COP) was in-situ synthesized on the surface of basalt fibers (BFs) for in-tube solid-phase microextraction (IT-SPME). The extraction tube, obtained via placing the modified BFs into a polyetheretherketone tube, was combined with high-performance liquid chromatography (HPLC) to achieve online IT-SPME-HPLC analysis. The important parameters, including sampling volume, sampling rate, organic solvent content and desorption time, were carefully investigated. Under the optimized conditions, the online analytical method was established for five estrogenic targets, with low limits of detection (0.001–0.005 μg/L), high enrichment factors (1800–2493), wide linear ranges (0.003–20, 0.015–20 μg/L) and satisfactory repeatability. It was successfully applied to detect five estrogens in a wastewater sample and a water sample in a polycarbonate cup. The BFs functionalized with COPs displayed excellent extraction effect for estrogenic pollutants, furthermore it has great potential in sample preparation or other fields.

## 1. Introduction

As one type of important organic pollutants, estrogenic compounds are associated with cancer, feminization, reproductive disorders and infertility [1,2]. In addition, estrogens were found in foods, cosmetics, sewage, surface water and even drinking water in many countries, and they can influence the normal activities of organisms by accumulating in the food chain [3,4,5,6]. Thus, it is very important to establish a sensitive and fast analytical method for trace estrogens in samples.

Due to the complexity, diversity of sample matrix and the trace level of targets, sample pretreatment has become an essential step during the analysis process. Some traditional methods, such as solid-phase extraction [7,8], stir bar sorptive extraction [9], liquid–liquid extraction [10,11,12,13], etc., have some disadvantages, like the consumption of a large volume of organic solvent, low extraction efficiency and poor sensitivity. Solid-phase microextraction (SPME) proposed by Pawlinszyn in 1989, not only overcomes the above shortcomings [14,15,16] but can also couple with gas chromatography (GC) [13,17], high-performance liquid chromatography (HPLC) [18] and other instruments. In particular, in-tube solid-phase microextraction (IT-SPME), which can be combined with HPLC [19,20,21], has been widely used in environmental, food, pharmaceutical and other analytical applications. For IT-SPME, the property of extraction materials directly affects the extraction performance and then influences the analytical results, so the extraction efficiency can be improved via selecting good extraction materials. So far, a variety of advanced materials have been used as the coatings for SPME, such as nanomaterials [22,23], carbon materials [24,25], mesporous materials [26,27], ionic liquids [28,29], aerogels [30,31], metal-organic frameworks [32], covalent organic frameworks [33], and so on. Covalent organic porous polymers (COPs) are completely composed of light elements (C, H, O, N, etc.) through covalent bonds, and have advantages such as controllable structure, adjustable function, good thermal and chemical stability, large specific surface, high porosity and strong adsorption [34,35]. COPs have become more and more popular in adsorption, separation and other fields in recent years. For example, Cheng’ group synthesized a new type of ionic COPs with gas selection holes, it gave a good gas adsorption performance and was successfully applied to the adsorption and separation of industrial CO_2_ [36]. Ma et al. prepared a mesoporous COP with hollow nanosphere structure, which was explored as the coating of SPME fiber to the detection of polycyclic aromatic hydrocarbons (PAHs) in environmental water samples, and satisfactory results were obtained [37].

In this paper, one COP was in-situ grown onto basalt fibers (BFs) as the extraction material for IT-SPME. COP-BFs were placed in a tube with 0.75 mm of inner diameter to prepare the extraction tube. The tube was coupled with HPLC to construct an online determination system of trace estrogenic targets. After the extraction and desorption conditions, including sampling rate, sampling volume, organic solvent content in sample and desorption time, were optimized, an online IT-SPME-HPLC method was established and applied to detect trace estrogens from real water samples.

## 2. Results and Discussion

### 2.1. Preparation of Extraction Tube

**Figure 1:**
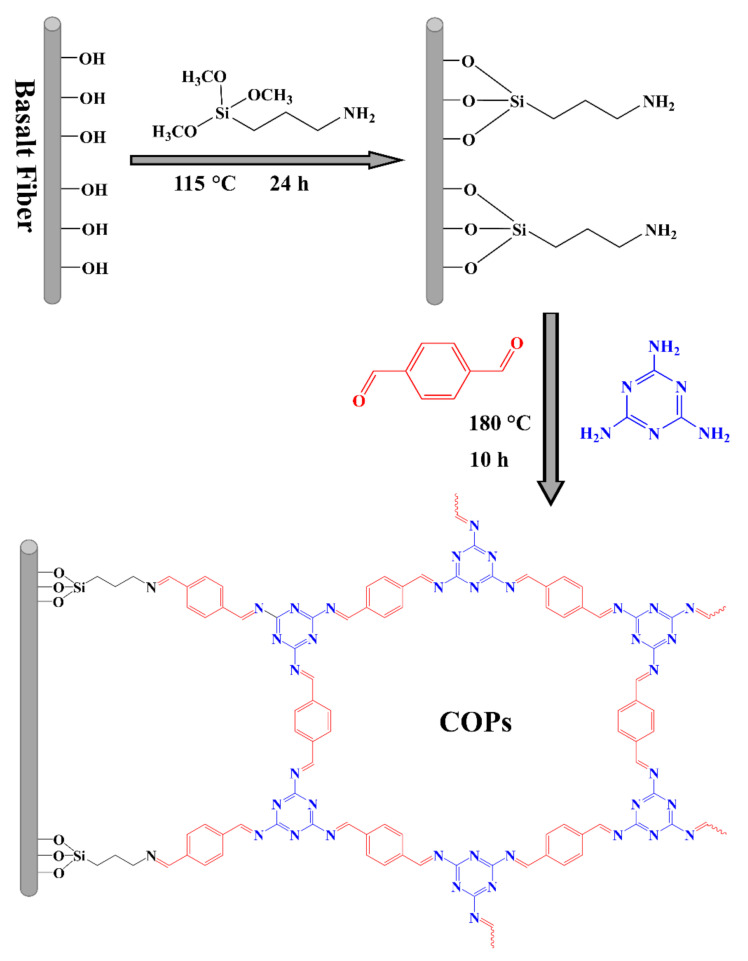
Schematic diagram of chemical reaction for in-situ generation of covalent organic porous polymers (COPs) on the surface of basalt fibers.

A bundle of BFs (0.22 g) was ultrasonically washed in acetone, ethanol and water for 30 min in turn. Then, it was dried at 50 °C under vacuum. BFs were immersed into 10 mL solution of 3-aminopropyltrimethoxysilane in toluene (20%). Under nitrogen atmosphere, the reaction solution was heated at 115 °C for 24 h. After that, the aminated BFs were washed in ethanol and water separately, and then dried at 80 °C in a vacuum oven. 0.47 g of melamine and 0.75 g of terephthalaldehyde were dissolved in 23 mL of dimethyl sulfoxide, and then the solution was transferred to a 100 mL high-pressure reactor, as well as the aminated BFs. The reactor was placed in an oven at 180 °C for 10 h. Finally, the fibers were taken out and washed by ethanol, and then dried under nitrogen atmosphere. The in-situ generation reaction of COPs on the surface of BFs is shown in Figure 1. The COPs-BFs (0.18 g) were filled into 30 cm length of polyetheretherketone (PEEK) tube. Another tube filled with the same amount of bare BFs was also prepared for comparison. Before use, the tubes were successively rinsed with 1.00 mL/min of ultrapure water and methanol for 1 h.

### 2.2. Characterization of Extraction Material

**Figure 2:**
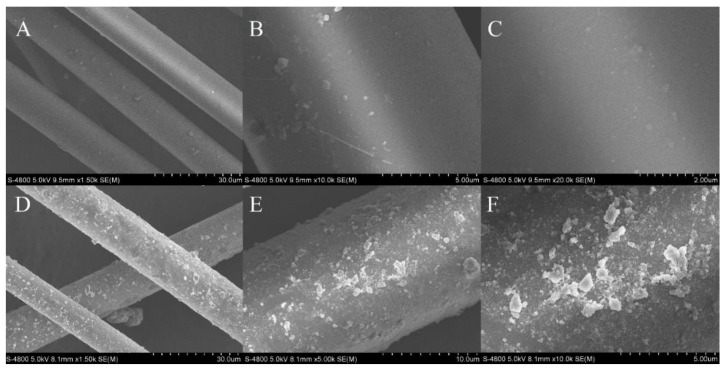
SEM diagrams of (**A**–**C**) bare basalt fibers and (**D**–**F**) basalt fibers functionalized with covalent organic porous polymers.

Bare BFs and COPs-BFs were characterized by scanning electron microscope (SEM). The surface of BFs (Figure 2A–C) is relatively smooth, but the surface of COPs-BFs (Figure 2D–F) is very rough. After the functionalization of COPs, a large number of irregular bulks were generated on the surface of BFs, which greatly increased the adsorption sites, facilitating the sufficient contact between targets and extraction material and improving the extraction ability and mass transfer efficiency.

**Figure 3:**
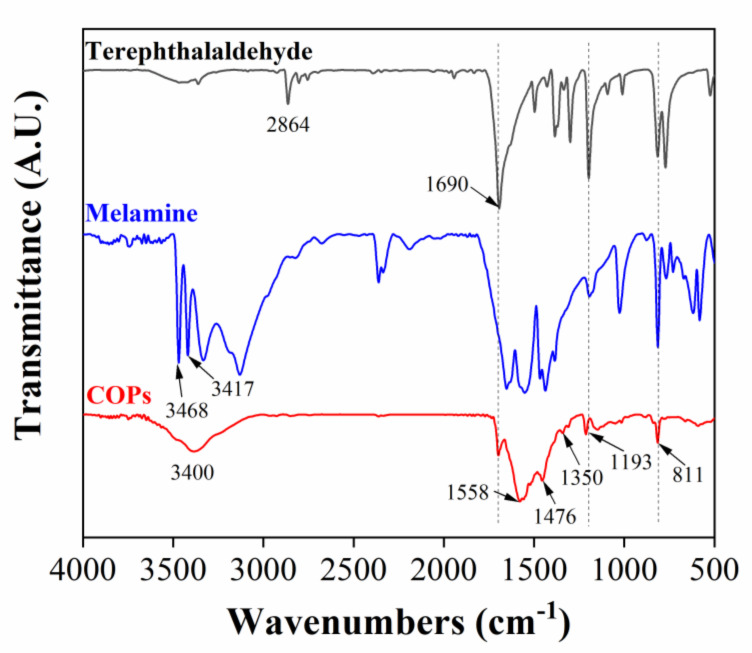
FT-IR spectra of COPs, melamine and terephthalaldehyde.

Fourier transform infrared (FT-IR) spectrum of COPs is shown in Figure 3. The disappearance of the primary amine group (3468 cm^−1^, 3417 cm^−1^) belonging to melamine indicates that melamine has been involved in the reaction. The disappearance of the C-H (2864 cm^−1^) vibration peak on terephthalaldehyde and the weakening of the C=O (1690 cm^−1^) stretching vibration peak indicate that terephthalaldehyde participates in the reaction. The peaks around 1558 and 1476 cm^−1^ are attributed to the C-N stretching vibration on the triazine ring. Meanwhile, the peaks at 1350 and 1193 cm^−1^ are caused by the C=N stretching vibration of melamine aromatic ring, and the peak at 811 cm^−1^ refers to the C-N bending vibration peak. These results further illustrate the successful synthesis of COPs.

### 2.3. Online IT-SPME-HPLC Procedure

According to our previous work [24], the extraction tube was connected to HPLC equipment for achieving the online IT-SPME-HPLC system. During the extraction, the six-port valve was set at load state, and the sample solution was transported through the tube by sampling pump. After extraction, the six-port valve was switched to injection state, and the mobile phase as desorption solvent flowed through the tube with a flow rate of 1.00 mL/min; at that moment, the analytes were desorbed from the extraction tube into the HPLC column and detector for the separation and detection. Subsequently, the six-port valve was transferred to the load state again for the next test.

### 2.4. Comparison of Extraction Performance between COPs-BFs and Bare BFs

**Figure 4:**
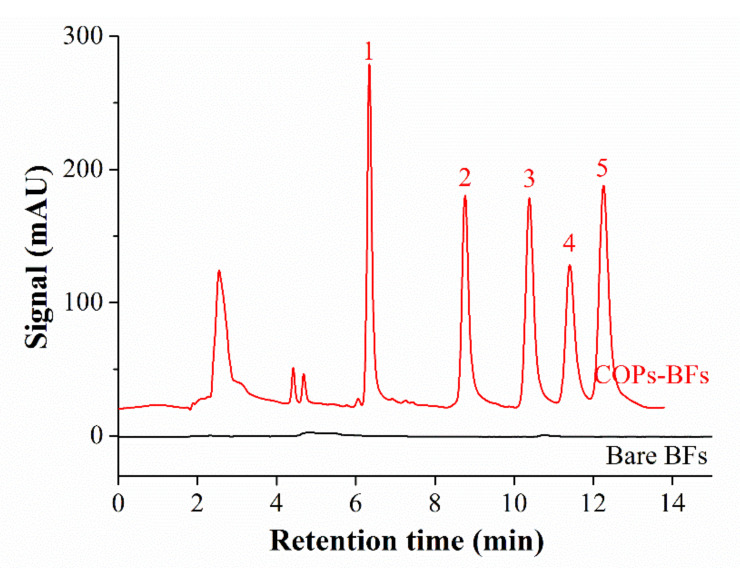
High-performance liquid chromatography (HPLC) chromatograms of 5 μg/L of standard samples after the extraction between the bare basalt fibers (BFs) tube and basalt fibers functionalized with covalent organic porous polymers (COPs-BFs) tube. Peaks: (1) bisphenol A, (2) 17 α-ethylestradiol, (3) estrone, (4) diethylstilbestrol, (5) hexestrol. Conditions: sampling volume, 60 mL; sampling rate, 1.50 mL/min; methanol content in sample, 1.0% (*v*/*v*); desorption time, 2.0 min; detection wavelength, 202 nm.

In order to confirm the contribution of COPs coating for extraction performance, the bare BFs tube was compared with COPs-BFs tube under the same conditions. As can be seen from Figure 4, there is no peak signal in the chromatogram on the bare BFs tube, that indicates almost no extraction ability. But the chromatographic peaks on the COPs-BFs tube are very obvious and even their heights are in the range of 100–250. These results proved that the extraction performance of the COPs-BFs tube was attributed from COPs coating.

### 2.5. Investigation of Extraction and Desorption Conditions

Five μg/L working solution of five estrogens was used to investigate the effect of sampling volume, sampling rate, organic solvent content in sample and desorption time on extraction efficiency.

**Figure 5:**
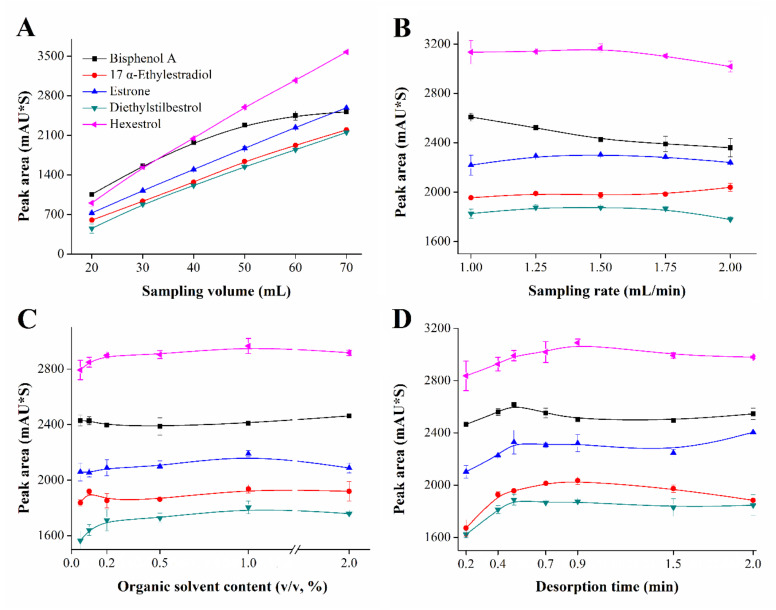
The images of extraction conditions optimization including (**A**) sampling volume, (**B**) sampling rate, (**C**) organic solvent content and (**D**) desorption time. Conditions: concentration of estrogens, 5 μg/L. The number of experiments to establish the plotted values was three (*n* = 3).

In in-tube SPME, sampling volume not only affects extraction efficiency but also decides analytical time. Too-large volume will prolong analysis time, which makes it difficult to achieve rapid analysis, but too-small volume may not achieve extraction equilibrium, leading to low extraction efficiency. The sampling volume was investigated from 20 to 70 mL; during this process, the sampling rate was fixed at 1.50 mL/min. As shown in Figure 5A, the growth trend of peak area is gradually weakening for bisphenol A with the increase of the volume, while the peak areas of the other four analytes show a straight growth. The adsorption saturation of bisphenol A will be reached with the increase of sampling volume, and competitive adsorption between other analytes and bisphenol A on the tube may also contribute to the result. Considering both extraction efficiency and analytical speed, 60 mL was selected as the optimal sampling volume.

The sampling rate has a significant influence on the extraction efficiency of IT-SPME. Low sampling rate is conducive to improve extraction efficiency, but extraction process is time-consuming. Although high sampling rate can shorten the analytical time, it reduces the contact time between the sample solution and extraction media, and it also raises the pressure in the tube. Therefore, it is necessary to find the optimal sampling rate to accomplish rapid analysis and improve extraction efficiency. In this work, the sampling rate was investigated, including 1.00, 1.25, 1.50, 1.75 and 2.00 mL/min. It can be seen from Figure 5B that the peak areas of bisphenol A, diethylstilbestrol and hexestrol slowly decrease with the increase of sampling rate, while 17 α-ethylestradiol and estrone are not significantly affected. In order to obtain satisfactory extraction efficiency and extraction speed, 1.50 mL/min was selected as the optimal sampling rate.

Since the extraction phase is Schiff base, increasing the content of organic solvent to an appropriate amount can inhibit the hydrolysis of the extraction phase and prolong the life. According to the thermodynamic theory of SPME, organic solvent can reduce the distribution coefficients of the analytes between extraction coating and sample solution through reducing the polarity of sample solution [38]. To investigate the effect of organic solvent, methanol as the organic solvent in the sample was controlled in the range of 0.05–2.0% (*v*/*v*). During the process, the sampling rate and volume were set as 1.50 mL/min and 60 mL, respectively. As shown in Figure 5C, the peak area of bisphenol A obviously decreases under more than 1.0% (*v*/*v*) of organic solvent, and the peak areas of the other four estrogens only show a slow decline. To achieve higher extraction efficiency and protect extraction phase, the content of organic solvent was selected to be 1.0% (*v*/*v*).

After extraction, the desorption is also critical for the accurate results. In this work, the extraction conditions were all in the optimal conditions, 1.00 mL/min of mobile phase (acetonitrile:water, 50:50, *v/v*) was directly used to desorb analytes from the tube, and the desorption time was selected from 0.2 to 2.0 min. As shown in Figure 5D, the peak areas of five estrogens raise from 0.2 to 0.5 min, but remain relatively stable at more than 0.7 min. Before the next test, the tube was also desorbed for 2.0 min to remove possible residual analytes on the tube. The residual was also investigated, and the results are shown in Supplementary Figure S1. In order to ensure more complete desorption and protect the extraction phase, the desorption time was set as 2.0 min for all tests.

After the investigation of the above parameters, the optimal conditions were finally confirmed as follows: 60 mL of sampling volume, 1.50 mL/min of sampling rate, 1.0% (*v*/*v*) of methanol in sample and 2.0 min of desorption time.

### 2.6. Method Evaluation

Under the optimal extraction and desorption conditions, the performances of the analytical method including linear ranges, linear coefficients, limits of detection (LODs) (three times of signal-to-noise ratio), enrichment factors and repeatability were investigated by a series of standard samples in deionized water, and the results are summarized in Table 1. The linear ranges of bisphenol A and hexestrol are 0.003–20 μg/L, and those of 17 α-ethylestradiol, estrone and diethylstilbestrol are 0.015–20 μg/L. The linear coefficients are higher than 0.9982, which means a good linear relationship. In addition, the LODs and enrichment factors of five estrogens are between 0.001–0.005 μg/L and 1800–2493 respectively, indicating great sensitivity and enrichment ability for trace targets. Total recoveries of five analytes ranging from 60.0% to 83.1% proved the high extraction efficiency of the tube. The relative standard deviations (RSDs, *n* = 3) of all analytes were less than 5.2%, which illustrates good extraction repeatability. The RSDs among three tubes ranged from 5.9% to 12.8%, the extraction tube has good reproducibility, and it can be easily obtained under the same preparation conditions.

### 2.7. Analysis of Real Samples

**Figure 6:**
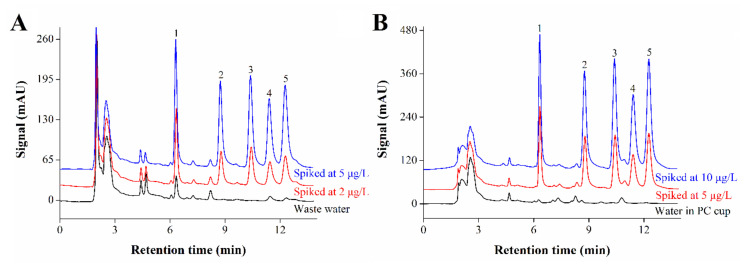
HPLC chromatograms of real water samples including (**A**) wastewater sample, spiked at 2 and 5 μg/L, and (**B**) water sample in polycarbonate (PC) cup, spiked at 5 and 10 μg/L. Peaks: (1) bisphenol A, (2) 17 α-ethylestradiol, (3) estrone, (4) diethylstilbestrol and (5) hexestrol. Conditions: sampling volume, 60 mL; sampling rate, 1.50 mL/min; methanol content in sample, 1.0% (*v*/*v*); desorption time, 2.0 min; detection wavelength, 202 nm.

In order to further prove the practicability of the analytical method, the method was used to detect the real samples containing wastewater and water in polycarbonate (PC) cup, and the relative recovery was also investigated and calculated. It can be seen from Figure 6 and Table 2 that four kinds of estrogens are detected from the wastewater, except for estrone. The concentrations of bisphenol A and diethylstilbestrol were determined as 0.24 and 0.39 μg/L, respectively. Two other targets were detected but not quantified. In the water sample in PC cup, only bisphenol A is detected but not quantified, and others could not be detected. The standard addition method was used to investigate the relative recovery in real samples. The recovery spikes at 2 and 5 μg/L were 84–106% and 88–98% in wastewater, and at 5 and 10 μg/L were 89–98% and 86–104% for the water sample in PC cup. According to the results, the analytical method is suitable for the detection of estrogenic compounds in real water samples. After more than 80 runs, the peak area remained at more than 90% of the original value for each analyte and there was only little loss of extraction efficiency for the tube, which indicated satisfactory durability of the tube. The cost of one extraction tube was less than fifteen dollars, which is cheap and acceptable for its real-world applicability.

## 3. Materials and Methods

### 3.1. Materials and Reagents

Bisphenol A, 17 α-ethylestradiol, estrone and melamine were acquired from Beijing J&K Scientific Ltd. (Beijing, China). Diethylstilbestrol and hexestrol were bought from Tokyo Pharmaron Industrial Co., Ltd. (Tokyo, Japan). Ethanol was acquired from Sinopharm Group Chemical Reagent Co., Ltd. (Shanghai, China). Dimethyl sulfoxide, 3-aminopropyltrimethoxysilane and terephthalaldehyde were purchased from Guangcheng Chemical Reagent Co., Ltd. (Tianjin, China). All above reagents were of analytical grade. Methanol and acetonitrile were of HPLC grade from America Tiandi Co., Ltd. (USA). Wastewater was collected from a local sewage plant. Polycarbonate (PC) cup was brought from a supermarket. Polyetheretherketone (PEEK) tube (0.75 mm inner diameter, 1.5 mm outer diameter) was obtained from Haohai Chemical Co. (Wuhan, China).

### 3.2. Apparatus

An Agilent 1260 HPLC system (Santa Clara, CA, USA) equipped with a 20 μL sample loop, a Zorbax C_18_ column (250 × 4.6 mm i.d., 5 μm) and a diode array detector (DAD) was used for the detection of estrogens. HPLC conditions included 25 °C of column temperature, 1.00 mL/min of acetonitrile:water (50:50, *v*/*v*) as mobile phase and the detection wavelength at 202 nm. A P600 pump from Beijing Laibo Taike Instrument Co., Ltd. (Beijing, China) was used to transport the sample solution through the extraction tube. Bare BFs and COPs-BFs were characterized by a field-emission scanning electron microscope (SEM, SUPRATM55, Carl Zeiss, AG, Germany). COPs were detected by a Fourier transform infrared spectrometer (FT-IR, NICOLET AVATAR 330, Thermo Electron Corporation, Waltham, MA, USA).

### 3.3. Preparation of Solution

The stock solution of five estrogens with a concentration of 10 mg/L was prepared with methanol and stored at 4 °C. The working solution was prepared daily in subsequent experiments. The boiling water was poured into a new PC cup, and then cooled to room temperature. Before the test, real water samples containing wastewater and water in a PC cup were filtered through a 0.45 µm membrane.

## 4. Conclusions

In this work, covalent organic porous polymers were in-situ prepared on the surface of basalt fibers, and an extraction tube packed with the functionalized fibers was developed. Combined with HPLC, an online analytical system was built. Using five estrogens as the targets, the extraction conditions were optimized, and an online analytical method was established. The method had low LODs, high enrichment factors, wide linear ranges and good repeatability. The method was further applied to the determination of trace estrogenic pollutants in some real water samples, and satisfactory results were obtained. Results indicated that the covalent organic porous polymers as extraction material exhibited good performance for SPME, and it has the potential to be used in other fields.

## Figures and Tables

**Table 1:** Analytical performances of the in-tube solid-phase microextraction (IT-SPME)-HPLC method for estrogens.

Analytes	Linear Ranges (μg/L)	^a^ r	LODs (μg/L)	Enrichment Factors	Total Recoveries	^b^ Extraction Repeatability (*n* = 3, RSD%)	^c^ Preparation Repeatability (*n* = 3, RSD%)
Bisphenol A	0.003–20	0.9985	0.001	1800	60.0%	1.9	12.8
17 α-Ethylestradiol	0.015–20	0.9986	0.005	2175	72.5%	5.2	8.4
Estrone	0.015–20	0.9982	0.005	2208	73.6%	4.3	8.0
Diethylstilbestrol	0.015–20	0.9993	0.005	2493	83.1%	4.5	5.9
Hexestrol	0.003–20	0.9990	0.001	2229	74.3%	2.6	7.0

^a^ Calibration level: *n* = 9. ^b^ Extraction repeatability was investigated by extracting 5 μg/L of estrogens standard aqueous solution three times. ^c^ Preparation repeatability was investigated by extracting 5 μg/L of estrogens standard aqueous solution through three tubes prepared under the same conditions.

**Table 2:** Analytical results and the recovery of five estrogens in two real samples.

Analytes	Wastewater (μg/L)	^a^ Recovery (*n* = 3, %)	^b^ Recovery (*n* = 3, %)	Water in PC cup (μg/L)	^b^ Recovery (*n* = 3, %)	^c^ Recovery (*n* = 3, %)
Bisphenol A	0.24 ± 0.02	102 ± 3.5	92 ± 3.2	NQ	98 ± 2.8	86 ± 3.8
17 α-Ethylestradiol	NQ	84 ± 3.8	96 ± 4.7	ND	91 ± 4.5	96 ± 4.0
Estrone	ND	86 ± 2.7	90 ± 5.6	ND	89 ± 3.9	97 ± 5.7
Diethylstilbestrol	0.39 ± 0.03	106 ± 1.4	98 ± 2.9	ND	94 ± 1.6	103 ± 4.1
Hexestrol	NQ	88 ± 4.6	88 ± 3.0	ND	93 ± 5.0	104 ± 2.2

ND is not detected. NQ is detected but cannot be quantified. ^a^ Standard addition level at 2 μg/L. ^b^ Standard addition level at 5 μg/L. ^c^ Standard addition level at 10 μg/L.

## Supplementary Materials

The following are available online. Figure S1: The effect of desorption time on the residual.
