# Supplementary material for: Application of Covalent Organic Porous Polymers-Functionalized Basalt Fibers for in-Tube Solid-Phase Microextraction
Source: Molecules. 2020 Dec 8;25(24):5788. doi: 10.3390/molecules25245788 (PMC7763957; doi:10.3390/molecules25245788)
Supplement: Supplementary file 1 [file molecules-25-05788-s001.pdf]

Supplementary Material

Application of Covalent Organic Porous Polymers-Functionalized Basalt Fibers for In-Tube Solid-Phase Microextraction

Qiong Jiang [1,*], Peng Xu [1], Juanjuan Feng [2] and Min Sun [2,*]

[1] College of Plant Protection, Gansu Agricultural University/Biocontrol Engineering Laboratory of Crop Diseases and Pests of Gansu Province, Lanzhou 730070, China; xupeng@gsau.edu.cn

[2] Key Laboratory of Interfacial Reaction & Sensing Analysis in Universities of Shandong, School of Chemistry and Chemical Engineering, University of Jinan, Jinan 250022, China; chm_fengjuanjuan@ujn.edu.cn

* Correspondence: menghuanrou@163.com (Q.J.); chm_sunm@ujn.edu.cn (M.S.); Tel.: +86-931-7632260 (Q.J.); +86-531-89736065 (M.S.)

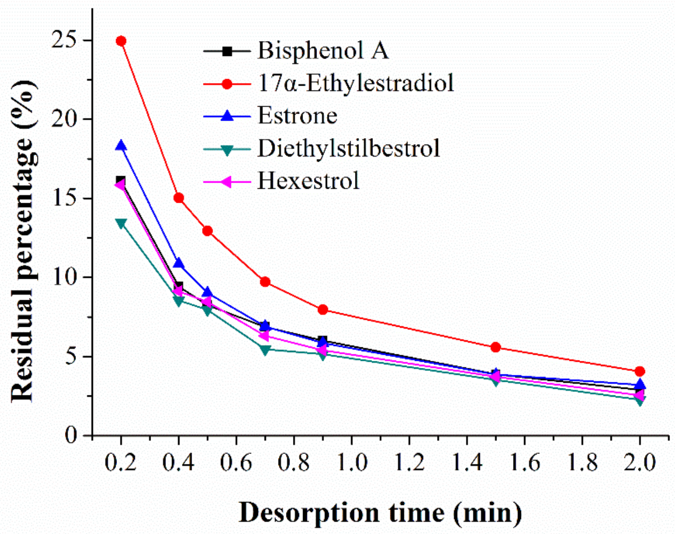

Figure S1. The effect of desorption time on the residual. Conditions: concentration of estrogens, 5 μg/L; sampling volume, 60 mL; sampling rate, 1.50 mL/min; methanol content in sample, 1.0% (*v*/*v*). Detection wavelength, 202 nm.
